# An Atypical Case of Myxedema Coma with Concomitant Nonconvulsive Seizure

**DOI:** 10.1155/2016/3438080

**Published:** 2016-10-30

**Authors:** Pratik Patel, Mikhael Bekkerman, Cristina Varallo-Rodriguez, Rajendra Rampersaud

**Affiliations:** ^1^Department of Medicine, St. John's Riverside Hospital, 967 N. Broadway, Yonkers, NY 10701, USA; ^2^Lake Erie College of Osteopathic Medicine, 1858 W Grandview Blvd, Erie, PA 16509, USA; ^3^Pulmonary and Critical Care, St. John's Riverside Hospital, 967 N. Broadway, Yonkers, NY 10701, USA

## Abstract

Hypothyroidism is a prevalent condition in the general population that is treatable with appropriately dosed thyroid hormone replacement medication. Infrequently, patients will present with myxedema coma, characterized by hypothermia, hypotension, bradycardia, and altered mental status in the setting of severe hypothyroidism. Myxedema coma has also been known to manifest in a number of unusual and dangerous forms. Here, we present the case of a woman we diagnosed with an uncharacteristic expression of myxedema coma and nonconvulsive seizure complicated by a right middle cerebral artery infarct.

## 1. Introduction

Hypothyroidism is a result of the inability of the thyroid gland to produce thyroid hormone sufficient enough to satisfy the requirements of peripheral tissues. Overt hypothyroidism is prevalent in the United States, hovering at 3.2–3.7% of the populations studied [1]. Clinical features of hypothyroidism can vary depending on a number of factors including age of onset and the severity of the disease and most commonly present as fatigue, lethargy, weakness, weight gain, cold intolerance, hair loss, depression, and xerosis. Measuring thyroid stimulating hormone (TSH) levels is the most appropriate method of diagnosing hypothyroidism. A thyroxine (T4) level should further be obtained if an abnormality is detected in the patient's TSH. A low level of T4 together with an elevated TSH confirms a diagnosis of hypothyroidism [2]. Severe hypothyroidism can manifest as myxedema coma, a rare, but frequently fatal disease characterized classically by hypothermia, bradycardia, hypotension, altered mental status, and cold intolerance. Several studies have shown that myxedema coma can present atypically, without the most common symptoms and clinical findings [3–7]. It is important for clinicians to be aware of the uncommon presentations of myxedema coma in order to avoid preventable complications.

## 2. Case Presentation

**Figure 1:**
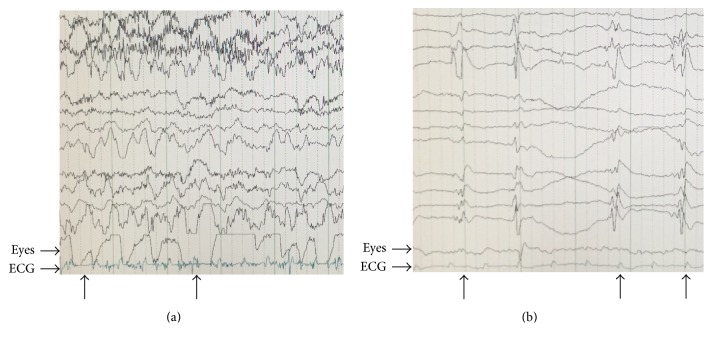
Electroencephalogram (EEG) displaying seizure-like activity at initial presentation (a) and at 10 days after admission (b). Arrows denote paroxysmal discharges.

A 70-year-old woman with a past medical history of hypothyroidism, coronary artery disease, cerebrovascular accident with right-sided weakness, and hypertension presented to the emergency department for altered mental status. Earlier that day, she had an appointment for a screening colonoscopy with her gastroenterologist. During the procedure, she was given propofol at 50 mg for sedation. The colonoscopy was complicated by stool in the rectum, and it was subsequently aborted. It was noted that her mental status was not improving after cessation of propofol, and thus the patient was sent to the emergency department. Vitals on arrival were notable for tachycardia at 120 bpm, temperature of 96.8°F (36°C), and blood pressure of 146/83 mm Hg. On examination, the patient was awake with her eyes open, but she was not responding to verbal stimuli. She reacted verbally and withdrew her extremities in response to pain, and her extremities were held in flexion. She scored an 11 on the NIH Stroke Scale. An ECG in the emergency department demonstrated normal sinus rhythm and a right bundle branch block. Laboratory studies on arrival revealed hyponatremia, hypokalemia, an elevated blood urea nitrogen (BUN) and creatinine, elevated glucose (298), elevated lactic acid (3.1), and an elevated thyroid stimulating hormone (TSH) at 175.0 mIU/mL. A CT of the head revealed no overt hemorrhage. An echocardiogram and magnetic resonance angiography study were performed to rule out a possible ischemic event, both of which resulted in no significant abnormalities. The patient was given an electroencephalogram (EEG), during which she developed left arm myoclonus. The EEG showed paroxysmal bursts of discharges suspect for complex partial seizures and wave complexes near the end of recording, suggesting nonconvulsive status epilepticus (Figure 1). The patient's son reported that she has no previous history of seizure activity. The patient received lorazepam 3 mg and placed on levetiracetam 1,500 mg and valproate 500 mg.

The patient was transferred to the intensive care unit and intubated the following day for airway protection after initiation of a midazolam drip. Due to the uncertain nature of her condition, we examined a broader differential diagnosis before we were confident with myxedema coma. The consulting endocrinologist initially began treatment with levothyroxine 25 mcg IV and hydrocortisone 100 mg three times daily. The midazolam drip was discontinued to assess the mental status of the patient, but the patient remained unarousable despite not being on any sedation medication. The nonconvulsive status epilepticus was treated with fosphenytoin 500 mg every 12 hours, lacosamide 200 mg twice a day, and levetiracetam 2000 mg twice a day. Approximately one week later, the patient's status epilepticus terminated and was noted with EEG. To assess for adrenal gland functionality, ACTH and AM cortisol levels were measured. The patient's ACTH level was found to be 49.9 pg/mL and her AM cortisol level was noted at 27.1 mcg/dL, not suggestive of any adrenal gland dysfunction. The patient remained intubated in the ICU for two weeks in a comatose state with occasional left sided myoclonus and bilateral upper and lower extremity nonpitting edema. She was unresponsive to verbal or painful stimuli. The patient's TSH and free thyroxine (T4) levels as measured in the ICU initially trended down but began to rise again after two weeks (Table 1).

The diagnosis of myxedema coma was made based on the patient's clinical presentation and a score of 65 on the diagnostic scoring system published in 2014 by Popoveniuc et al. [8]. The typical characteristics of myxedema coma, including bradycardia, hypotension, and hypothermia, were absent in this case. However, a score of >60 using these criteria is reported to be diagnostic of myxedema coma with a sensitivity of 100%. The patient's dose of levothyroxine was increased to 50 mcg IV, 25 mcg IM, and 25 mcg of liothyronine (T3) in the ICU and subsequently levothyroxine was increased to 50 mcg bid IV and 50 mcg of liothyronine due to the later increase in the patient's TSH levels.

**Figure 2:**
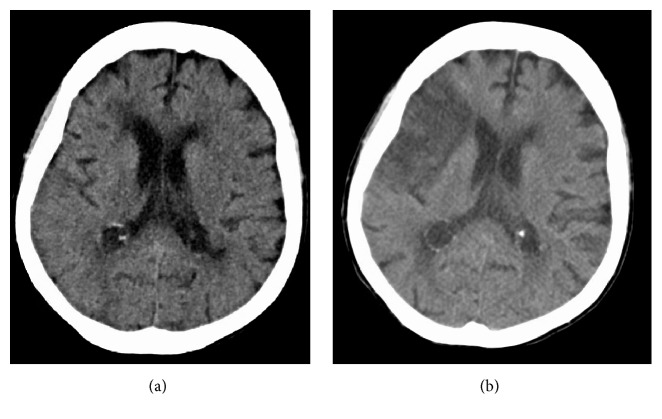
CT scan of the head performed on admission (a) compared to one performed after 14 days in the ICU (b) shows development of a large right MCA infarct.

A repeat CT of the head was performed after 14 days in the ICU, which revealed a large, nonhemorrhagic middle cerebral artery (MCA) infarct that was not present on admission (Figure 2). On the same hospital day, the patient became arousable, and she was able to follow basic verbal commands. She had marked weakness of the left lower extremity and absent motor function of the left upper extremity. A tracheostomy was performed due to depressed respiratory function and inability to wean off ventilator support. The patient was scheduled to undergo a percutaneous endoscopic gastrostomy (PEG) tube insertion and subsequent transfer to a long-term rehabilitation facility to help improve her motor function as well as her respiratory function.

## 3. Discussion

Myxedema coma is a rare endocrine emergency due to the advent of rapid TSH ELISA testing but has been reported to occur with an incidence of 220,000 per year in western countries [9]. The mortality rate is reported to be 60%. It typically presents as a state of severe hypothyroidism with cold intolerance, hypothermia, hypoventilation, hypercapnia, bradycardia, and cardiomegaly. Oftentimes there is a precipitating factor such as infection, trauma, medication, CVA, or a metabolic abnormality [8]. In our case, the precipitating factor was likely the propofol administration during the patient's colonoscopy. The patient's myxedema coma together with her epileptic activity likely resulted in cerebral vasoconstriction and subsequently hypoperfusion to her brain, precipitating the right middle cerebral artery infarction. It is important to suspect myxedema coma in patients with a history of hypothyroidism presenting with altered mental status after a known precipitating event and to begin empiric treatment with large doses of synthetic T4 (200–400 mcg) and T3 promptly to avoid complications [3]. A swift diagnosis can be critical to a patient's prognosis. There are several published criteria available that allow physicians to make the diagnosis of myxedema coma [8, 10]. Additionally, we recommend ruling out seizure in a patient that presents in a newly onset obtunded or comatose state due to risk of cerebral hypoperfusion and ischemic infarct.

## Figures and Tables

**Table 1:** Measured TSH and free thyroxine (T4) levels during hospitalization. Normal values for TSH range between 0.358 and 3.74 mIU/mL and free T4 range between 0.76 and 1.46 ng/dL.

Day of hospitalization	1	3	5	9	11	13	17
TSH level (mIU/mL)	175.00	35.70	7.53	20.10	18.10	30.90	50.70
Free T4 level (ng/dL)	0.58	0.75	0.56	0.37	0.38	0.47	0.69
